# Familial Alzheimer’s Disease and Recessive Modifiers

**DOI:** 10.1007/s12035-019-01798-0

**Published:** 2019-10-29

**Authors:** Jorge I. Vélez, Francisco Lopera, Claudia T. Silva, Andrés Villegas, Lady G. Espinosa, Oscar M. Vidal, Claudio A. Mastronardi, Mauricio Arcos-Burgos

**Affiliations:** 1 Universidad del Norte, Barranquilla, Colombia; 2 Neuroscience Research Group, University of Antioquia, Medellín, Colombia; 3 INPAC Research Group, Fundación Universitaria Sanitas, Bogotá, Colombia; 4 Grupo de Investigación en Psiquiatría (GIPSI), Departamento de Psiquiatría, Instituto de Investigaciones Médicas (IIM), Facultad de Medicina, Universidad de Antioquia, Medellín, Colombia

**Keywords:** Alzheimer’s disease, *PSEN1*, Age of onset, Recessive Mutations, Genetic Isolates, Genetic Interactions

## Abstract

Alzheimer’s disease (AD) is progressive brain disorder that affects ~ 50 million people worldwide and has no current effective treatment. AD age of onset (ADAOO) has shown to be critical for the identification of genes that modify the appearance of AD signs and symptoms in a specific population. We clinically characterized and whole-exome genotyped 71 individuals with AD from the *Paisa* genetic isolate, segregating the (*PSEN1*) E280A dominant fully penetrant mutation, and analyzed the potential recessive effects of ~ 50,000 common functional genomic variants to the ADAOO. Standard quality control and filtering procedures were applied, and recessive single- and multi-locus linear mixed-effects models were used. We identified genetic variants in the *SLC9C1*, *CSN1S1*, and *LOXL4* acting recessively to delay ADAOO up to ~ 11, ~ 6, and ~ 9 years on average, respectively. In contrast, the CC recessive genotype in marker *DHRS4L2*-rs2273946 accelerates ADAOO by ~ 8 years. This study, reports new recessive variants modifying ADAOO in *PSEN1* E280A mutation carriers. This set of genes are implicated in important biological processes and molecular functions commonly affected by genes associated with the etiology of AD such as *APP*, *APOE*, and *CLU*. Future functional studies using modern techniques such as induced pluripotent stem cells will allow a better understanding of the over expression and down regulation of these recessive modifier variants and hence the pathogenesis of AD. These results are important for prediction of AD and ultimately, substantial to develop new therapeutic strategies for individuals at risk or affected by AD.

**Electronic supplementary material:**

The online version of this article (10.1007/s12035-019-01798-0) contains supplementary material, which is available to authorized users.

## Introduction

The global incidence and prevalence of Alzheimer’s disease (AD) are increasing at alarming rates. Without intervention, 1 in 85 people worldwide will develop AD by 2050 [1]. Remarkably, the delay of the AD age of onset (ADAOO) by 1 year would result in ~ 9 million fewer cases of the disease worldwide by 2050 [2]. Therefore, it is imperative to advance our efforts in therapeutic and preventative strategies, not only to cure, but also to delay the ADAOO.

Several studies show that linear and non-linear gene variant interactions modify the ADAOO affecting significantly the cognitive decline, changing the AD natural history, and delaying the ADAOO even by more than a decade [3–10]. Genetic epistasis (non-linear interaction among genes) is shaped by the genetic structure of the population and consequently by the individual genomic structure. In the case of genetic isolates, the steady increase in homozygosis (deficit of heterozygosis) not only outlines and defines population micro differentiation (substructure), but plays a pivotal role in shaping individual complex phenotypes [11]. In this vein, it was around 30 years ago that our group first made the clinical and genetic characterization of the world-over largest pedigree segregating AD ascertained from a genetic isolate. More than 5,000 individuals grouped into 25 families, half of whom will develop AD at an early age, constitute this pedigree [6, 12]. Most people in this pedigree live in and around Yarumal, a town engraved in the Andean mountains of Northeast Colombia. People inhabiting this region call themselves “*Paisas*” [11]. Individuals belonging to the “*Paisa* pedigree,” affected by AD, carry a deleterious variant in the Presenilin 1 (*PSEN1*) gene and most will develop dementia before their 50th birthday (we have coined the term the *Paisa* pedigree, making reference to the clan cluster with this homogeneous form of AD) [12]. This variant, a G**A**A[Glu]➔ G**C**A[Ala] substitution at position 73,664,808 in chromosome 14, is often referred to as the E280A or *Paisa* mutation.

The founder effect of this pedigree dates from the time Spanish Conquistadors colonized Colombia during the 16th century [6, 12]. To date, individuals grouped into these families are recognized as descendants of the original founder [11]. This pedigree is a unique resource for neurological and genetic research into AD as it contains exhaustive and detailed medical records from thousands of individuals, with multiple and prospective follow-ups, including neurological evaluations, neuropsychological tests, biomarkers, and image data [13].

Alzheimer’s disease recurs within families more often than expected by chance alone. It is well accepted that AD follows an autosomal dominant pattern of inheritance, especially in families of the *Paisa* community suffering from the most severe form of AD [14]. However, several studies have also dissected mutations and duplications, particularly in the Apoliprotein (*APP*) gene, that act in a recessive fashion to dramatically change AD susceptibility [15–19]. Recessive contributions can be particularly inferred in populations exhibiting high degrees of consanguinity and higher prevalence of disease than the general population [20] such as the *Paisa* genetic isolate in Colombia [11] and the Wadi Ara population in Israel [21, 22]. However, there is no evidence of the potential role that these, nor other recessive mutations, may play as ADAOO modifiers in individuals with AD carrying the E280A mutation.

In previous reports, we tested the influence of dominant major genes interacting with the *PSEN1*-E280A mutation to modify ADAOO. However, the presence of recessive interactions of functional variants on ADAOO is yet to be explored. This is legitimate problem, given (1) the significant inbreeding that is present in this genetic isolate and (2) the increasing evidence that recessive mutations may be important in AD neuropathology. In this manuscript, we tackle the hypothesis that the homozygosis structure at the individual level in carriers of the E280A mutation underpins the major gene recessive epistasis modifying the ADAOO. Given that genes encoding enzymes and blood-transported substrates act recessively, the definition of such themes might be suitable to outline potential therapeutic targets.

## Subjects and Methods

### Patients, DNA Extraction, and Genotyping

Detailed clinical, paraclinical, and ascertainment procedures, applied to this pedigree, have been presented elsewhere [12, 23, 24]. The Ethics Committee of the University of Antioquia approved this study (Protocol 1115- 408-20543). Informed consent was obtained from all participants. A total of 71 individuals with the E280A mutation were included for the analysis. Genomic DNA was extracted from peripheral blood from all patients and processed by the Australian Genome Facility (Melbourne, VIC, Australia). Seventy-one individuals with AD from the E280A pedigree were genotyped; 57 were subject to whole-exome genotyping (WEG) using Illumina® HumanExome BeadChip-12v1_A and 14 underwent whole-exome capture (WEC). A detailed description of the genotyping, capture, and WEG and WEC methods has been presented elsewhere [5]. Samples with SNPs call rates below Illumina’s® expected 99% were excluded.

### Genetic, Statistical, and Bioinformatics Analyses

#### Quality Control, Filtering, and Classification of Functional Variants

After importing the genetic data to Golden Helix’s® SVS 8.3.0, a single genetic data file was constructed by merging the exonic variants from both the WEG and WEC platforms. Genotypes for 71 individuals from the E280A pedigree were obtained, and quality control subsequently performed using the following exclusion criteria: (*i*) deviations from Hardy-Weinberg equilibrium with *P* values < 0.05/*m* (where *m* is the number of markers included for analysis); (*ii*) genotype call rate < 90%; (*iii*) presence of one or > 2 alleles. Genotype and allelic frequencies were estimated by maximum likelihood. Following previous recommendations [25], variants with a minor allele frequency (MAF) ≥ 0.01 were classified as common and as rare otherwise. Rare variants were excluded from the analysis. Exonic variants with potential functional effect were determined using the functional prediction information available in the dbNSFP_NS_Functional_Predictions GRCh_37 annotation track [26]. The dbNSFP is an integrated database of functional annotations from multiple sources for the comprehensive collection of human non-synonymous SNPs (NSs). Its current version includes a total of 82,832,027 NSs and splice site SNPs, and compile prediction scores from 14 prediction algorithms including SIFT, Polyphen2, LRT, MutationTaster, MutationAssessor, FATHMM, MetaSVM, MetaLR, VEST, PROVEAN, FATHMM-MKL coding and fitCons, eight conservation scores (phyloP46way_primate, phyloP46way_placental, phyloP100way_vertebrate, phastCons46way_primate, phastCons46way_placental, phastCons100way_veterbrate, GERP++ and SiPhy), and other function annotations [27–29]. This filter is fully implemented in the Golden Helix® SVS 8.3.0 Variant Classification module. This module was also used to examine interactions between variants and gene transcripts to classify variants based on their potential effect on genes. Variants were classified according to their position in a gene transcript. In addition, variants in coding exons were further classified according to their effect on the gene’s protein sequence.

#### GWAS Analysis of Common Variants

We studied the association between common exonic functional variants (CEFVs) and ADAOO using single- and multi-locus recessive linear mixed-effect models (LMEMs) [30] with up to 10 steps in the backward/forward optimization algorithm. Both types of models are implemented in Golden Helix® SVS 8.3.0. The advantage of these models is the inclusion of both fixed (genotype markers, sex, and years of education) and random effects (family or population structure); the latter to account for potential inbreeding by including a kinship matrix (that is, the identity-by-descent [IBD], which in our case was estimated between all pairs of individuals using markers excluded from the final analysis after linkage disequilibrium [LD] pruning).

A single-locus LMEM assumes that all loci have a small effect on the trait, while a multi-locus LMEM assumes that several loci have a large effect on the trait [30]. The optimal model was selected using a comprehensive exploration of multiple criteria including the Extended Bayes Information Criteria (*eBIC*)*,* the Modified Bayes Information Criteria (*mBIC*), and the Multiple Posterior Probability of Association (*mPPA*). After the estimation process using the forward/backward algorithm concluded, the estimated coefficients ${\hat{\beta}}_1,{\hat{\beta}}_2,\dots, {\hat{\beta}}_m$were extracted and a hypothesis test of the form *H*_0,*i*_: *β*_*i*_ = 0 vs. *H*_1,*i*_: *β*_*i*_ ≠ 0 was performed for the *i*th CEFVs to obtain the corresponding *P* value, *P*_*i*_ (*i* = 1,2,…,*m*). The collection *P*_1_, *P*_2_,…, *P*_*m*_ was subsequently corrected for multiple testing using the false discovery rate (FDR) [31, 32] using R [33].

#### Biological Relatedness Between Candidate and Core Genes

We used the Human Gene Connectome (HGC) database [34] to quantify the biological relatedness between ADAOO modifier genes identified in our set of *PSEN1* E280A mutation carriers and genes reported to cause this form of AD. The HGC is more effectively applied when seeking to identify Mendelian disease-causing genes. Thus, using the HGC database could disclose some relevant information pointing cryptic networks out from the genes reported by our GWAS analysis of common variants (see “Subjects and Methods” section). The rationale of the HGC is to prioritize candidate genes on the basis of their functional relevance to the AD phenotype. Candidate genes were chosen on the basis of their quantitative relatedness or biological distance to genes already established as having functional importance in AD. Biological distances [34] were calculated between genes identified in our association analyses and those previously identified in AD. To evaluate the significance of these distances, *P* values are estimated via random permutation of pairwise gene interactions in the HGC database.

## Results

### Subjects

Demographic data, including age, ADAOO, gender, and schooling of individuals with familial and sporadic AD have been presented comprehensively in previous manuscripts [3, 5]. Briefly, 71 patients with AD carrying the E280A mutation and from the extremes of the ADAOO distribution (44 women [62%] and 27 men [38%]) were clinically evaluated and genotyped using either WEG or WEC (see “Methods” section). Years of education ranged from 0 to 19 years (6 ± 4.24 years, *n* = 57); 4 (7%) individuals never attended school, and 28 (49.1%), 21 (36.9%), and 4 (7%) attended but not necessarily completed elementary school (grades 1 to 5), high school (grades 6 to 11, inclusive), and tertiary education, respectively. The ADAOO (mean ± SD) was 47.8 ± 5.8 years, with no statistically significant differences by gender (female 47.6 ± 6.1; male 48.4 ± 5.5, *P* = 0.55) and education groups (*F*_3,53_ = 2.721, *P* = 0.053).

### Quality Control and Genetic Population Structure

**Fig 1:**
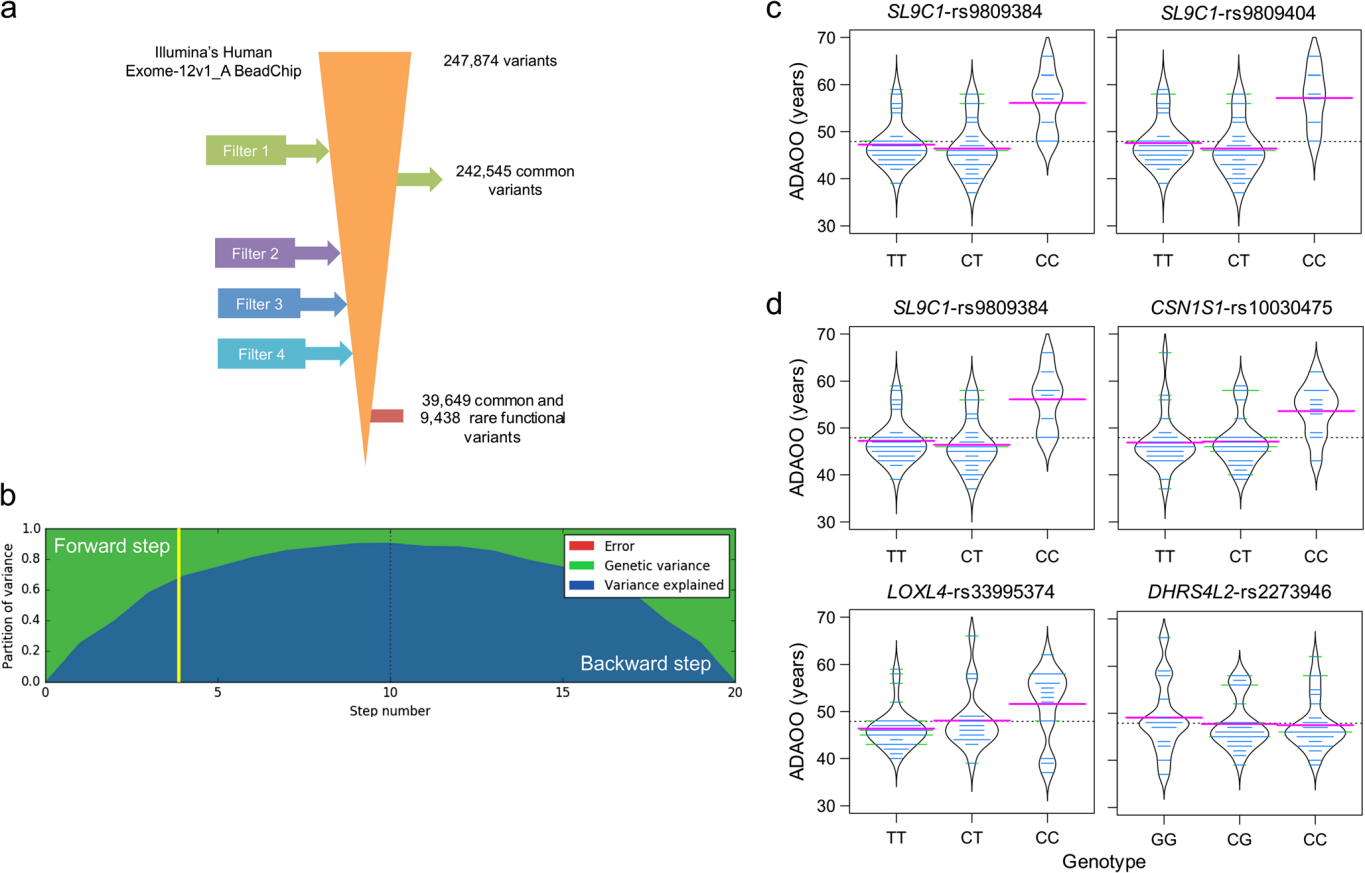
**a** Filtering process applied to exonic variants. *Filter 1* includes common variants between the Illumina’s Human Exome-12V1_A BeadChip and the whole-exome capture. *Filter 2* excludes variants with a genotype call rate < 90%, in Hardy-Weinberg disequilibrium and with one or > 2 alleles. *Filter 3* excludes variants with MAF < 1% and *Filter 4* excludes those nonfunctional. A total of 49,087 CEFVs remained for analysis. **b** Partition of phenotypic variance for each forward inclusion (steps 1 to 10) and backward elimination (10 steps after the dotted line). The yellow vertical line marks the selected model based on the highest multiple posterior probability of association (*mPPA*). Beanplots for ADAOO as a function of genotypes in variants with *P*_FDR_ < 0.05 when **c** single- and **d** multi-locus linear mixed-effects models were used (see Table 1). Pink, blue, and dotted horizontal lines correspond, respectively, to the within genotype average ADAOO, the individuals’ ADAOO, and the global average ADAOO in our sample of 71 *PSEN1* E280A mutation carriers. ADAOO, Alzheimer’s disease age of onset; CEFVs, common exonic functional variants

After quality control, assembling, and filtering, a total of 49,087 common and rare variants with potential functional effects remained for genetic analyses (Fig. 1a). To control for potential genetic population stratification (population subdivision), we estimated the *F*_st_ statistic of S. Wright. The *F*_st_ value is 0.0187 for our cohort, which suggests no micro-differentiation.

### Recessive Variants Modifying ADAOO

A recessive multi-locus LMEM with four steps in the backward/forward optimization algorithm was selected as the optimal model best explaining the ADAOO variance (> 65% in total; Fig. 1b). The application of a single-locus LMEM reported that markers *SLC9C1-*rs9809384 and *SLC9C1-*rs9809404, separated by 46 base pairs and in full linkage disequilibrium, delayed ADAOO ($\hat{\beta}>0$; Table 1a and Fig. 1c). Interestingly, marker *SLC9C1-*rs9809384 (*P* = 11.03 × 10^−15^; *P*_FDR_ = 4.05 × 10^−11^) was also found to be statistically significantly associated as an ADOO modifier when a multi-locus LMEM was used, suggesting that its effect on ADAOO withstands the presence of other interacting CEFVs modifying the history of disease. Under this recessive model, the non-synonymous mutations *CSN1S1-*rs10030475 (CC genotype, *P* = 4.76 × 10^−9^, *P*_FDR_ = 5.43 × 10^−5^), *LOXL4*-rs33995374 (CC genotype, *P* = 3.28 × 10^−7^, *P*_FDR_ = 0.0024), and *DHRS4L2*-rs2273946 (CC genotype, *P* = 3.48 × 10^−7^, *P*_FDR_ = 1.9 × 10^−3^) were also found to be ADAOO modifiers in our cohort of individuals with AD carrying the *PSEN1* E280A mutation (Table 1b). Because all estimated *β* coefficients are positive, the presence of two copies of the alternate allele in markers harbored in the *SLC9C1*, *CSN1S1*, and *LOXL4* genes delays ADAOO up to ~ 11, ~ 6, and ~ 9 years on average, respectively ($\hat{\beta}>0$; Table 1b and Fig. 1d). In contrast, having the CC genotype in *DHRS4L2*-rs2273946 accelerates ADAOO by ~ 8 years, on average ($\hat{\beta}<0$; Table 1b and Fig. 1d). No gender- or education-specific effects of these SNPs were found (Table 2).

**Table 1:** Results of the association analysis using recessive (a) single- and (b) multi-locus linear mixed-effect models for ADAOO in 71 patients with *PSEN1* E280A Alzheimer’s disease

(**a**)										
**Chr**	**SNPa**	**Position**	**Gene**	**Marker Information**				**Single-locus linear mixed-effects model**		
				**Ref/Alt**	**MAF**	**CR**	**Change**	$\hat{\beta}\left({\mathrm{SE}}_{\hat{\beta}}\right)$	***P***	***P***_**FDR**_
3	rs9809384	111,981,878	*SL9C1*	T/C	0.32	1	p.Ile364Val	9.73 (1.61)	8.06 × 10^−8^	1.8 × 10^−3^
3	rs9809404	111,981,924	*SL9C1*	T/C	0.29	1	p.Ile348Met	10.85 (1.80)	8.13 × 10^−8^	9.2 × 10^−4^
(**b**)										
**Chr**	**SNPa**	**Position**	**Gene**	**Marker Information**				**Multi-locus linear mixed-effects model**		
				**Ref/Alt**	**MAF**	**CR**	**Change**	$\hat{\beta}\left({\mathrm{SE}}_{\hat{\beta}}\right)$	***P***	***P***_**FDR**_
3	rs9809384	111,981,878	*SLC9C1*	T/C	0.32	1	p.Ile364Val	11.03 (1.06)	1.77 × 10^−15^	4.05 × 10^−11^
4	rs10030475	70,807,771	*CSN1S1*	C/T	0.42	0.97	p.Ala117Val	6.37 (0.94)	4.76 × 10^−9^	5.43 × 10^−5^
10	rs33995374	100,020,880	*LOXL4*	C/T	0.2	0.98	p.Arg154Gln	8.80 (1.55)	3.28 × 10^−7^	2.4 × 10^−3^
14	rs2273946	24,458,162	*DHRS4L2*	G/C	0.32	0.98	p.Gln2His	-8.13 (1.43)	3.48 × 10^−7^	1.9 × 10^−3^

^a^UCSC GRCh37/hg19 coordinates. *AOO*, age of onset*; Chr*, chromosome; *SNP*, single-nucleotide polymorphism; *Ref/Alt*, reference/alternate allele; *MAF*, minimum allele frequency; *CR*, call rate; $\hat{\beta}$, regression coefficient; ${\mathrm{SE}}_{\hat{\beta}}$, standard error of $\hat{\beta}$; *P* , *P* value; *FDR*, false discovery rate. Highlighted variants accelerate ADAOO

**Table 2:** Gender- and education-specific effects of associated SNPs on ADAOO

Chr	SNPa	Sex			Education groupb		
		χ^2^	df	*P*	*χ*^2^	df	*P*
3	rs9809384	2.532	2	0.282	7.373	4	0.117
3	rs9809404	1.269	2	0.530	7.373	4	0.117
4	rs10030475	0.255	2	0.880	2.538	4	0.638
10	rs33995374	1.239	2	0.538	3.317	4	0.506
14	rs2273946	1.239	2	0.538	3.317	4	0.506

^a^UCSC GRCh37/hg19 coordinates

^b^Includes “no,” “primary,” “middle,” and “tertiary” education

*ADAOO*, Alzheimer’s disease age of onset; *Chr*, chromosome; *SNP*, single-nucleotide polymorphism. *χ*^2^, test statistic; *df*, degrees of freedom

### Biologically Related Genes

We successfully identified seven statistically significant biological relatednesses between previously reported genes conferring susceptibility to AD and those found to be recessive ADAOO modifiers in our cohort (Table 3). Of particular importance are the pairwise comparisons involving *APOE*, and those where *APP* is involved because of their effects on ADAOO and AD susceptibility. Additional interpretations are provided in the Supplementary Material.

**Table 3:** Statistically significant biological relatedness between AD core genes and those (target genes) identified as ADAOO modifiers

AD gene	Target	Distancea	***P***	Route
*APOE*	*CSN1S1*	6.0	0.0109	*APOE* [1.25] *CSNK2A1* [1.76] *CSN1S1*
*CLU*	*CSN1S1*	7.0	0.0226	*CLU* [1.755] *CUL1* [1.74] *CSN1S1*
*APP*	*DHRS4L2*	10.2	0.0274	*APP* [1.74] *UBC* [3.38] *DHRS4L2*
*APP*	*LOXL4*	10.2	0.0279	*APP* [1.74] *UBC* [3.38] *LOXL4*
*CLU*	*SLC9C1*	17.6	0.0289	*CLU* [1.25] *HRAS* [1.25] *BRAP* [3.37] *SLC9C1*
*APOE*	*DHRS4L2*	10.2	0.0329	*APOE* [1.74 ]*UBC* [3.38] *DHRS4L2*
*APOE*	*LOXL4*	10.2	0.0335	*APOE* [1.74] *UBC* [3.38] *LOXL4*

^a^Calculated as in Itan et al*.* [34]. *AD*, Alzheimer’s disease; *APOE* = apolipoprotein E; *APP*, amyloid beta precursor protein; *BRAP*, BRCA1 associated protein; *CLU,* clusterin; *CSN1S1*, casein alpha S1; *CSNK2A*1, casein kinase 2 alpha 1; *DHRS4L2*, dehydrogenase/reductase 4 Like 2; *HRAS*, HRas proto-oncogene, GTPase; *LOXL4*, lysyl oxidase like 4; *SLC9C1*, solute carrier family 9 member C1; *UBC*, ubiquitin C; *P*, *P* value. Values within [] correspond to the direct biological distance between genes. For more details, see the “Materials and Methods” section in [34]

## Discussion

Here we show an important set of genes whose role in the pathophysiology and therapeutics of Alzheimer’s disease (AD) could be further investigated. Individuals included in this study exhibit an extreme phenotype, that is, suffer from AD caused by the *PSEN1* E280A fully penetrant mutation, belong to the Paisa genetic isolate and their AD age of onset (ADAOO) ranges from the early 30s to the late 70s [23]. In previous studies [3, 5, 7, 35], we successfully applied this sampling strategy using extreme phenotypes (of major effect phenotypes) [36–39]. Power estimates are included in the Supplementary Material. Briefly, using the current sample size and testing *m* = 100,000 common exonic functional variants, a number that certainly exceeds the number of variants tested in this study, yields to a *post hoc* power estimate > 99%.

We identified that variants harbored within the Solute Carrier Family 9 Member C1 (*SLC9C1*), the Casein Alpha S1 (*CSN1S1*), and the Lysyl Oxidase Like 4 (*LOXL4*) genes delay the AD age of onset (ADAOO) up to ~ 11 years. In contrast, a non-synonymous variant harbored in the Dehydrogenase/Reductase 4 Like 2 (*DHRS4L2*) gene accelerates ADAOO up to ~ 8 years (Table 1). To the best of our knowledge, this is the first time that variants within the genes reported herein either delay or accelerate ADAOO under a recessive oligogenic model in individuals with familiar AD caused by a fully penetrant mutation.

The *SLC9C1* gene, located at 3q13.2, has a cell membrane sub-cellular localization, and has been related with an ion channel activity and maintenance of the acid-base homeostasis [40]. Molecules with this role have been linked with several pathologies, such as cancer, where there is an aberrant regulation of hydrogen ion dynamics leading to pH disruption in a variety of cells and tissues. Genes such as *SLC9C1* have been use to downregulate pH regulation capacity on cancer cells [41]. SLC9C1 is expressed in the brain, the cortex, and the cerebellum [42]. We hypothesized that, because of its antiporter activity, *SLC9C1* could enable the balance of the cell/tissue pH and influence the transport of solutes and molecules across the membrane, cleaning up the debris or surplus molecules deposited in E280A affected neurons, allowing them to survive longer due perhaps to the positive activity of *SLC9C1*. Moreover, its ortholog―*DNhe2* in *Drosophila―*has shown to control intracellular pH and to induce adult epithelial and embryonic stem cell differentiation [43], an important feature that enable cell renewal that could potentially induced cell-replacement in affected cells.

Gene ontology analyses indicate that *CSN1S1* plays an important role in the capacity of milk to transport calcium phosphate. It is intriguing that *CSN1S1* is biologically related to the Apolipoprotein E (*APOE*) and Clusterin (*CLU*) genes (Table 2), which have been shown to delay ADAOO in *PSEN1* E280A mutation carriers and as a mediator of Aβ toxicity with neuroprotective effects, respectively [5, 35, 44]. Epidemiological studies suggest that intake of milk and fermented dairy products are significantly associated with a decreased risk of AD, cognitive decline, and cognitive-related disorders in the elderly [45–49]. A mouse model of AD fed with camembert cheese, which was obtained from sterilized milk fermented with *Lactococcus lactis* to reduce the pH and subsequently with *Penicillium candidum*, showed both significantly reduced levels of amyloid β (Aβ) accumulation and hippocampal inflammation, and enhancing hippocampal neurotrophic factors [50]. These results provide supporting biological and molecular evidence on the preventive effects of milk-derived products on AD susceptibility, which were reported only epidemiologically. Finding that *PSEN1* mutation carriers with the CC recessive genotype in *CSN1S1-*rs10030475 have a ~ 6-year delay on the ADOO (Table 1 and Fig. 1d) sheds some light into developing new therapeutic alternatives against AD focused on nutrigenomic research [51–53].

*LOXL4* belongs to a family of five copper-dependent amine oxidases including *LOX*, *LOXL, LOXL2*, *LOXL3*, and *LOXL4*. These genes have been shown to be involved in extracellular matrix (ECM) formation, founding the crosslinking between collagen and elastin [54]. *LOXL4* has been reported to be responsible for the lysine-derived cross-links toward collagen and elastin, essential in biogenesis of extracellular matrix. However, the LOX family has several other functions important in cancer progression such as cell growth, cell adhesion, migration, and invasion [55]. Perhaps, *LOXL4* proliferative migratory activity could influence *PSEN1*-affected cells and modify their ECM components through the activation of Src/FAK signaling axis, which controls epithelial morphology [55, 56]. LOXL4 ECM induced plasticity as well as induction of proliferative activity could lead to a better physiological stability of *PSEN1*-mutated cells, which results in the ADAOO delaying effect observed in these patients. This effect is in line with the delayer effect of the APOE*E2 allele in this cohort [5, 35]. Given the biological relatedness between *APOE* and *LOXL4*(Table 3), the synergistic effect of these two genes on ADAOO is yet to be elucidated.

A non-synonymous variant within the *DHRS4L2* gene that accelerates ADAOO was identified (rs2273946, *P* = 3.48 × 10^−7^, *P*_FDR_ = 1.9 × 10^−3^; Table 1). This result suggests that *DHRS4L2* activity in *PSEN1* mutation carriers is directly associated with alterations of the proteolytic γ-secretase subunit, which is fundamental to cleave many transmembrane proteins. *DHRS4L2* is expressed in the brain―the cortex and the cerebellum―[42] and belongs to the SDR enzyme family. They have shown to play a role in the NAD/NADP-dependentdehydrogenase-reductases(NRDR) activity on a large and heterogeneous set of substrates including steroids, retinoid, prostaglandins, metabolites, and xenobiotics [57]. We hypothesize that, as a consequence of such alterations, more Aβ plaques accumulate in the CNS and neurons apoptosis is subsequently initiated. Thus, misregulation of *DHRS4L2* could exacerbate the AD phenotype, ultimately leading to the appearance AD sign and symptoms at early ages in *PSEN1* mutation carriers. DHRS4L2 enzyme has been shown to reduce endogenous products, such as prostaglandins, biogenic aldehydes, steroids, reactive lipid peroxidation products, and xenobiotic compounds (i.e., pharmacologic drugs, carcinogens, and toxicants), suggesting that NRDR may be implicated in the metabolism and detoxification of carbonyl compounds [58]. Action of dehydrogenase alteration has also been linked to neurodegeneration process, due to increased toxic aldehydes. Such dysregulation in dehydrogenase activity has shown to increase cytotoxicity, oxidative stress, energy deficits, apoptosis, and cell death [59]. Interestingly, Li et al. (2012) [58] found a natural antisense transcript, the long noncoding RNA (lncRNA) AS1DHRS4, which lies on the opposite strand of *DHRS4*, and the 5′ ends of the genes overlap. The authors showed that silencing *AS1DHRS4* resulted in both increased mRNA and protein expression of DHRS4, and increased mRNA expression of *DHRS4L1* and *DHRS4L2.* The control exerted by this lncRNA on *DHRS4L2* highlights the importance of this gene in the homeostasis and cell balance in AD. Moreover, the biological relatedness between *DHRS4L2* and *APP*(Table 3), a gene harboring recessive mutations causing AD [15–19], reinforces the importance of further studying the accelerating effect on the ADAOO of this variant within *DHRS4L2* in other AD populations.

Considering that no current effective treatment to slow or even stop AD progression is available, human-induced pluripotent stem cells (iPSCs), which have greatly facilitated the generation of patient-specific neurons to study patient-specific characteristics in AD [60], represent a new way of assessing the patient-specific basis of disease and what the effect would be when particular changes are introduced. Future validation studies of our findings are needed. Such studies, we propose, will greatly benefit from using iPSCs coming from *PSEN1* E280A mutation carriers [61] to investigating the over expression and downregulation of some of these ADAOO recessive modifier variants and better understand the pathogenesis of the AD in our patients, as well as the potential development of new therapeutic targets for AD.

In summary, here we identified recessive mutations shaping the natural history of AD in members of a multigenerational extended family carrying the *PSEN1* E280A mutation. In this regard, it is important to highlight that, because the set of individuals carrying the *PSEN1* E280 mutation in this study and those analyzed in Vélez et al [5] are the same, both studies are highly correlated. However, in the current study, we explore whether recessive variants may explain ADAOO variability. As demonstrated by this study, new important themes, once missed by the dominant and codominant of transmission, were strongly associated to the ADAOO. Although previous studies have provided conclusive evidence that recessive genetic variations within increase AD susceptibility and cause AD in Caucasian and Japanese populations [15–19], our results suggest that-high order recessive genetic interactions between *PSEN1* and the set of genes reported herein have a significant modifier effect on ADAOO in individuals with AD from the Paisa genetic isolate.

## Electronic supplementary material

ESM 1 (DOCX 598 kb)
