# Supplementary material for: Familial Alzheimer’s Disease and Recessive Modifiers
Source: Mol Neurobiol. 2019 Oct 29;57(2):1035–43. doi: 10.1007/s12035-019-01798-0 (PMC7031188; doi:10.1007/s12035-019-01798-0)
Supplement: Supplementary file 1 — (DOCX 598 kb) [file 12035_2019_1798_MOESM1_ESM.docx]

**Supplementary Material for:**

**Familial Alzheimer’s Disease and Recessive Modifiers**

Jorge I. Vélez^1,*^, Francisco Lopera^2,*^, Claudia T. Silva^3^, Andrés Villegas^1^, Lady G. Espinosa^4^,
Oscar M. Vidal^1^, Claudio A. Mastronardi^3,*^, Mauricio Arcos-Burgos^4,*^

September 10, 2019

1. *Universidad del Norte, Barranquilla, Colombia.*
2. *Neuroscience Research Group, University of Antioquia, Medellín, Colombia.*
3. *INPAC Research Group, Fundación Universitaria Sanitas, Bogotá, Colombia.*
4. *Grupo de Investigación en Psiquiatría (GIPSI), Departamento de Psiquiatría, Instituto de Investigaciones Médicas (IIM), Facultad de Medicina, Universidad de Antioquia, Medellín, Colombia.*

*^*^These authors contributed equally to this work.*

^#^ Correspondence to be directed to:

Professor Mauricio Arcos-Burgos, MD, PhD.

Grupo de Investigación en Psiquiatría (GIPSI),

Departamento de Psiquiatría,

Instituto de Investigaciones Médicas (IIM),

Facultad de Medicina,
Universidad de Antioquia,

Medellín, Colombia.

E-mail: [mauricio.arcos@udea.edu.co](mailto:mauricio.arcos@udea.edu.co)

**Supplementary Table 1.** Gene ontology (GO) processes for the network involving recessive ADAOO modifier genes. *P*_FDR_ = *P-*value corrected by multiple testing using False Discovery Rate.

| **Term ID** | **Term description** | **Genes involved** | | | | ***P*_FDR_** |
| --- | --- | --- | --- | --- | --- | --- |
|  |  | ***APOE**** | ***PSEN1*** | ***SLC9C1**** | ***LOXL4**** |  |
| GO:1905908 | positive regulation of amyloid fibril formation |  |  |  |  | 0.0011 |
| GO:0042982 | amyloid precursor protein metabolic process |  |  |  |  | 0.0039 |
| GO:0060999 | positive regulation of dendritic spine development |  |  |  |  | 0.0140 |
| GO:1903793 | positive regulation of anion transport |  |  |  |  | 0.0185 |
| GO:0050818 | regulation of coagulation |  |  |  |  | 0.0320 |
| GO:0032092 | positive regulation of protein binding |  |  |  |  | 0.0350 |
| GO:0007613 | memory |  |  |  |  | 0.0413 |
| GO:0010977 | negative regulation of neuron projection development |  |  |  |  | 0.0413 |
| GO:0030003 | cellular cation homeostasis |  |  |  |  | 0.0413 |
| GO:0043524 | negative regulation of neuron apoptotic process |  |  |  |  | 0.0413 |
| GO:0098657 | import into cell |  |  |  |  | 0.0413 |
| GO:0098771 | inorganic ion homeostasis |  |  |  |  | 0.0413 |
| GO:1903364 | positive regulation of cellular protein catabolic process |  |  |  |  | 0.0413 |
| GO:0048167 | regulation of synaptic plasticity |  |  |  |  | 0.0433 |
| GO:0050770 | regulation of axonogenesis |  |  |  |  | 0.0433 |
| GO:0050808 | synapse organization |  |  |  |  | 0.0464 |
| GO:0006898 | receptor-mediated endocytosis |  |  |  |  | 0.0497 |

* Age of onset decelerators. See Table 1

GO analysis were performed using STRING V11. For more information see <https://version-11-0.string-db.org/cgi/network.pl?networkId=8I3oXiwyHRhx>

**Oligogenic model for detecting ADAOO modifiers**

Following a similar approach to that in Vélez et al. 2016 (Vélez et al, 2016), we conducted a power analysis using the pwr (Champely, 2015) package in R (R Core Team, 2015) suggests that, for a *k*=3 group design, 71 individuals would be sufficient to detect 80% true positives and a large effect (defined by the Cohen’s *d* parameter; *d*=0.82, Supplementary Figure 1) when *m=*100,000 variants are tested for association (a value that certainly overcomes the final number of variants used during the LMEM analyses). The selection of such effect is based on our hypothesis that variants of large effect (i.e., mutations) recessively modify ADAOO in *PSEN1* E280A mutation carriers (see also the $\hat{\beta}$ coefficients in Table 1 of the main text); *k* is selected based on the maximum number of possible genotypes for a biallelic genetic marker; and *m*, as mentioned before, to be conservative.

**Supplementary Figure 1**. **(a)** Sample size (*n*) as a function of the Cohen’s effect size (*d*) and power. The type I error probability used for calculations is 0.05/100,000. In **(b)** the blue dot corresponds to *n=*71, power = 80% and a large effect *d =* 0.82.

Here, *d* implies that *d*% of the ADAOO variance is explained by the *m* independent variables (i.e., ~100,000 genetic variants being tested). Our LMEMs estimates show that the percentage of ADAOO variance explained with only four CEFVs is > 65% for the *n*=71 individuals (Table 1b). Under these conditions (*d=*0.65, *n=*71, *m=*100,000), the *post hoc* power estimate is >99%, which slightly increases when four CEFVs are included instead.

**References**

1. Vélez JI, Lopera F, Sepulveda-Falla D, *et al. APOE*E2* allele delays age of onset in PSEN1 E280A Alzheimer's disease. Mol Psychiatry. 2016 Jul;21(7):916-24. doi: 10.1038/mp.2015.177.
2. Champely S. pwr: Basic Functions for Power Analysis. R package version 1.1-2. URL: <http://CRAN.R-project.org/package=pwr>. 2015.
3. R Core Team. R: A language and environment for statistical computing. R Foundation for Statistical Computing, Vienna, Austria. URL: <http://www.R-project.org>. 2015.
